# Synthesis, Characterization, Antibacterial Properties, and In Vitro Studies of Selenium and Strontium Co-Substituted Hydroxyapatite

**DOI:** 10.3390/ijms22084246

**Published:** 2021-04-19

**Authors:** Muhammad Maqbool, Qaisar Nawaz, Muhammad Atiq Ur Rehman, Mark Cresswell, Phil Jackson, Katrin Hurle, Rainer Detsch, Wolfgang H. Goldmann, Asma Tufail Shah, Aldo R. Boccaccini

**Affiliations:** 1 Institute of Biomaterials, University of Erlangen-Nuremberg, 91058 Erlangen, Germany; maqboolchoudary@gmail.com (M.M.); qaisar.nawaz@fau.de (Q.N.); atique1.1@hotmail.com (M.A.U.R.); rainer.detsch@fau.de (R.D.); 2 Lucideon Ltd., Penkhull, Stoke-on-Trent, Staffordshire ST4 7LQ, UK; Mark.Cresswell@lucideon.com (M.C.); Phil.Jackson@lucideon.com (P.J.); 3 CAM Bioceramics B.V., 2333 CL Leiden, The Netherlands; 4 Department of Materials Science and Engineering, Institute of Space Technology Islamabad, Islamabad 44000, Pakistan; 5 GeoZentrum Nordbayern, Mineralogy, University of Erlangen-Nuremberg, 91054 Erlangen, Germany; katrin.hurle@fau.de; 6 Department of Biophysics, University of Erlangen-Nuremberg, 91052 Erlangen, Germany; wgoldmann@biomed.uni-erlangen.de; 7 Interdisciplinary Research Centre in Biomedical Materials (IRCBM), COMSATS University Islamabad Lahore Campus, Defence Road, Off-Raiwind Road, Lahore 54000, Pakistan; drasmashah@cuilahore.edu.pk

**Keywords:** co-substituted hydroxyapatite, strontium, selenium, antibacterial, cytocompatibility

## Abstract

In this study, as a measure to enhance the antimicrobial activity of biomaterials, the selenium ions have been substituted into hydroxyapatite (HA) at different concentration levels. To balance the potential cytotoxic effects of selenite ions (SeO_3_^2−^) in HA, strontium (Sr^2+^) was co-substituted at the same concentration. Selenium and strontium-substituted hydroxyapatites (Se-Sr-HA) at equal molar ratios of *x* Se/(Se + P) and *x* Sr/(Sr + Ca) at (*x* = 0, 0.01, 0.03, 0.05, 0.1, and 0.2) were synthesized via the wet precipitation route and sintered at 900 °C. The effect of the two-ion concentration on morphology, surface charge, composition, antibacterial ability, and cell viability were studied. X-ray diffraction verified the phase purity and confirmed the substitution of selenium and strontium ions. Acellular in vitro bioactivity tests revealed that Se-Sr-HA was highly bioactive compared to pure HA. Se-Sr-HA samples showed excellent antibacterial activity against both Gram-negative (*Escherichia coli*) and Gram-positive (*Staphylococcus carnosus*) bacterial strains. In vitro cell–material interaction, using human osteosarcoma cells MG-63 studied by WST-8 assay, showed that Se-HA has a cytotoxic effect; however, the co-substitution of strontium in Se-HA offsets the negative impact of selenium and enhanced the biological properties of HA. Hence, the prepared samples are a suitable choice for antibacterial coatings and bone filler applications.

## 1. Introduction

Hydroxyapatite (HA) (Ca_10_(PO_4_)_6_(OH)_2_) is a versatile solid biomaterial with a stoichiometric composition similar to natural bone and a Ca/P ratio of 1.67. It is the most extensively used bioceramic owing to its excellent biocompatibility and bioactivity as well as osteoconductive properties [1]. Based on its viscous nature in cement form and excellent compressive strength, HA has been widely used in orthopedic and maxillofacial applications to support bone growth and remodeling [2]. HA has also been widely utilized in drug delivery applications [3], bioactive coating material on metallic osseous implants [4,5], for cancer treatment [6], and as a filler for bio-composites and scaffolds [7,8]. Natural apatite has better physical and biological properties compared to synthetic hydroxyapatite due to a significant amount of ion-substituted impurities [9], and therefore, increasing research is being carried out to enhance the physical and biological properties of HA by incorporating different elements in its framework [10,11,12]. In HA, both anions and cations can be substituted with other metal ions [11,13,14]. Metal substitution changes the physical (i.e., crystallinity, lattice parameters, and stability) as well as the biological properties of HA [15]. For example, silicon has been found to enhance the bone mineralization of HA [16], while magnesium (Mg) improves its bioactivity [17].

Bacterial infections affect a large number of implants after implantation, despite the improvement of biomaterials. Metal ion-doping in bioceramics represents an important approach to significantly reduce the bacterial infection, particularly in bone-related implants. Silver (Ag) and zinc (Zn) have been found to induce antimicrobial properties in HA [18,19]. Selenium is an important nutrient present in biological tissues, and selenium-containing biomaterials have been shown to retard bone tumors and improve the biological activity of tissues [20]. Selenium also reveals antioxidant behavior and shields the human body against free radicals and carcinogens. A study by Ravi et al. [21] revealed that strontium-substituted HA (10 wt % Sr-HA) exhibited a reduction in microbial growth of ≈56% for *E.coli* and 35% for *S. aureus*. Uskokovic et al. [22] showed that the selenite ions can easily replace phosphate ions in the HA lattice structure because the size of the selenite ion is almost identical to that of the phosphate ion, and selenium-substituted HA showed improved anticancer and antibacterial effects compared to pure HA. However, the use of selenium is limited by its potentially high cytotoxic effect. It is hypothesized that the co-substitution of a second ion may help in compensating the side effects caused by the first element and facilitates the incorporation of two different ions by balancing the ionic radii or valence. For instance, the addition of Sr^2+^ has been found to reduce the cytotoxic effects of silver ions and also to improve the cell proliferation and differentiation properties of HA. Liu et al. [23] found that Sr^2+^ incorporation in coralline HA significantly improves osteo-conductivity and inductivity. Wei et al. [24] reported the synthesis of hydroxyapatite co-doped with selenium and strontium ions, but they did not study the antibacterial properties. Zhang et al. [25] synthesized Si-Sr co-substituted HA and reported that compared to mono-substituted Si-HA and Sr-HA, di-substituted HA had better biological properties and the ability to stimulate cell attachment and proliferation of osteoblasts and mesenchymal stem cells. Thus, strontium ions possess osteo-conductive and progenic properties, which are significant in the bone regeneration process.

Here, we are reporting the synthesis of novel selenium and strontium co-substituted hydroxyapatite by a co-precipitation method. The objective of the co-substitution was to use antibacterial properties of selenium against Gram-negative (*Escherichia coli*) and Gram-positive (*Staphylococcus carnosus*) bacteria and to substitute strontium, aiming at offsetting the cytotoxic effect of selenium and enhancing cell viability and bioactivity. Selenite has a structural similarity with phosphate tetrahedral, and therefore, it is an ideal ion for ionic substitution. Strontium is in the same alkaline earth group with calcium and both are divalent ions. The prepared selenium and strontium co-substituted HA is anticipated to be an ideal bioceramic for dental and biomedical applications, as a carrier for drugs and genes, and especially as a coating material for implants, where the infection is of significant concern.

## 2. Results

### 2.1. Material Characterization

#### 2.1.1. XRD Analysis

**Figure 1:**
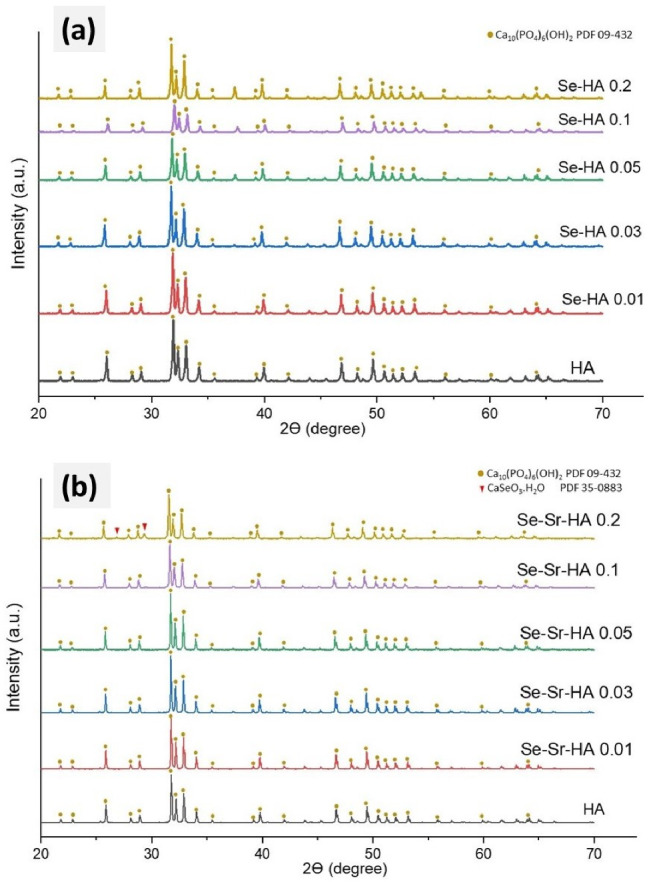
XRD patterns of selenium-substituted hydroxyapatite (Se-HA) (**a**) and selenium and strontium-substituted hydroxyapatites (Se-Sr-HA) with different Se and Se/Sr molar ratios (**b**) sintered at 900 °C.

Figure 1a,b presents powder X-ray diffractograms of selenium-substituted hydroxyapatite (Se-HA) and selenium strontium co-substituted hydroxyapatite (Se-Sr-HA) samples at different Se/(Se + P) and Sr/(Sr + Ca) ratios. All diffractograms corresponding to HA, Se-HA, and Se-Sr-HA were highly crystalline, and their characteristic peaks matched the standard diffractogram of hexagonal HA (space group P63/m) (PDF 09-432). The characteristic diffraction peaks for hexagonal HA were obvious in both Se-HA and Se-Sr-HA samples. The results revealed that no major secondary phases were detected in samples substituted with lower concentrations of SeO_3_^2−^ and Sr^2+^ ions. Small traces of CaSeO_3_.H_2_O and CaO were detected in the diffractogram of Se-Sr-HA 0.2. Remarkably, a slight shifting in diffraction peaks toward a lower angle (2θ) compared to the reference HA peaks was observed, which is due to ionic substitution in the HA lattice [26]. The crystallite sizes of undoped as well as SeO_3_^2−^ and Sr^2+^-substituted samples were all above 500 nm; therefore, no meaningful data could be obtained by Rietveld refinement. The microstrain decreased for samples with lower dopant contents: while a microstrain of 0.14 ± 0.01 was obtained for HA, the values were around 0.08–0.09 for samples Se-Sr-HA 0.01 to Se-Sr-HA 0.1. For Se-Sr-HA 0.2, the microstrain increased to 0.18 ± 0.01.

The lattice parameters of Se-HA revealed that the a-axis values were increased, while c-axis values were reduced as in the case of Se-HA. Both lattice parameters in Se-Sr-HA were enlarged with increasing Se and Sr concentration when compared to those of the reference HA. This is likely to be due to the larger ionic radius of Sr^2+^ (118 pm) compared to Ca^2+^ (99 pm), so that Sr^2+^ expands the size of the HA unit cell. The unit cell volume experienced a corresponding increase with increasing lattice parameters, as demonstrated in Table 1.

SeO_3_^2−^ has a different structure (flat trigonal pyramid) than PO_4_^3−^ ions, leading to a considerable effect on a-axis lattice parameters, regardless of the similar ionic radii to that of PO_4_^3−^ ions [27]. On the other hand, the substitution of tetrahedral SeO_4_^2−^ having extensively larger ionic radii did not affect the dimensions of the lattice structure [27]. In the studies by Ma et al. [28], an increase in Se/P ratio did not reveal any significant change in the a-axis; however, a reduction in the c-axis was observed.

#### 2.1.2. Fourier Transform Infrared Spectroscopy (FTIR)

**Figure 2:**
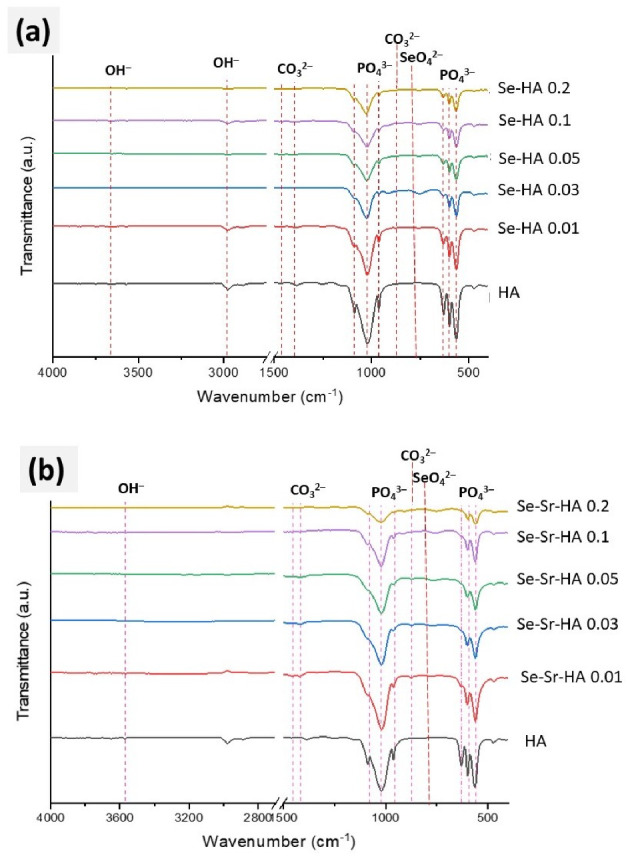
FTIR spectra of Se-HA (**a**) and Se-Sr-HA (**b**) at different Se and Se/Sr molar ratios sintered at 900 °C.

FTIR spectra of pure HA, Se-HA *x* (where *x* = 0.01, 0.03, 0.05, 0.1 and 0.2), and Se-Sr-HA *x* (where *x* = 0.01, 0.03, 0.05, 0.1 and 0.2) samples are presented in Figure 2a,b. The characteristic peaks of asymmetric stretching for a PO_4_^3−^ group in HA were observed in the range of 900 to 1200 cm^−1^ [28]. The transmission bands of the triply degenerated stretching mode of the P-O bond of the phosphate group were observed in all samples at 1087 cm^−1^ (ν_3a asym._) and 1046–1032 cm^−1^ (ν_3b asym._ and ν_3c asym._, shoulder peak) [27]. The ν_3b_ and ν_3c_ merged to give a broad peak in all samples. The peak at 962 cm^−1^ in HA was due to the non-degenerated stretching mode (ν_1 sym._) of the P-O bond in the PO_4_^3−^ group [27]. Two sharp peaks (ν_4a_), (ν_4c_) and one shoulder peak (ν_4b_) (almost merged with ν_4c_) were characteristic of triply degenerated bending mode of O-P-O of PO_4_^3−^ group and were observed at 601 cm^−1^ (ν_4a_), 574 cm^−1^ (ν_4b_) and 560 cm^−1^ (ν_4b_) [29].

In the Se-Sr-HA sample, the intensity of all above peaks was reduced, particularly the symmetric and asymmetric stretching bands, with increasing value of Sr/(Sr + Ca) and Se/(Se + P) due to the substitution of a PO_4_^3−^ ion with a selenite ion [28]. Similar changes were observed for transmission bands for O-P-O bending mode, at 601 cm^−1^ and 560 cm^−1^ [28], where peak intensities were reduced with increasing strontium content. However, no changes in peak position, for both bending and stretching bands, were observed in the case of selenite substitution. The peak at 631 cm^−1^ in HA was due to librational mode, ν_L_, of the hydroxyl group [30]. This peak was absent in Se-HA and Se-Sr-HA, as reported by Boyd et al., due to the replacement of carbonate at OH^−^ sites [31]. In HA, the small band at 1405 cm^−1^ was characteristic of the stretching mode (ν_3_) in CO_3_^2−^. However, there was an obvious shift in the frequency of other peaks, as reported by Bigi et al. [32] for strontium substitution, which might be due to co-substituted selenium ions. In Se-HA and Se-Sr-HA, a few weak peaks observed at 767 cm^−1^ were attributed to the bending vibration of SeO_4_^2−^ tetrahedron [33]. The intensity of the first peak decreased with an increase in Se/P ratio, while the intensity of the bending peak increased with increasing Se/P or Se/(Se + P) ratio [28]. These results may indicate the substitution of metal ions in the HA sample.

#### 2.1.3. Raman Spectroscopy

**Figure 3:**
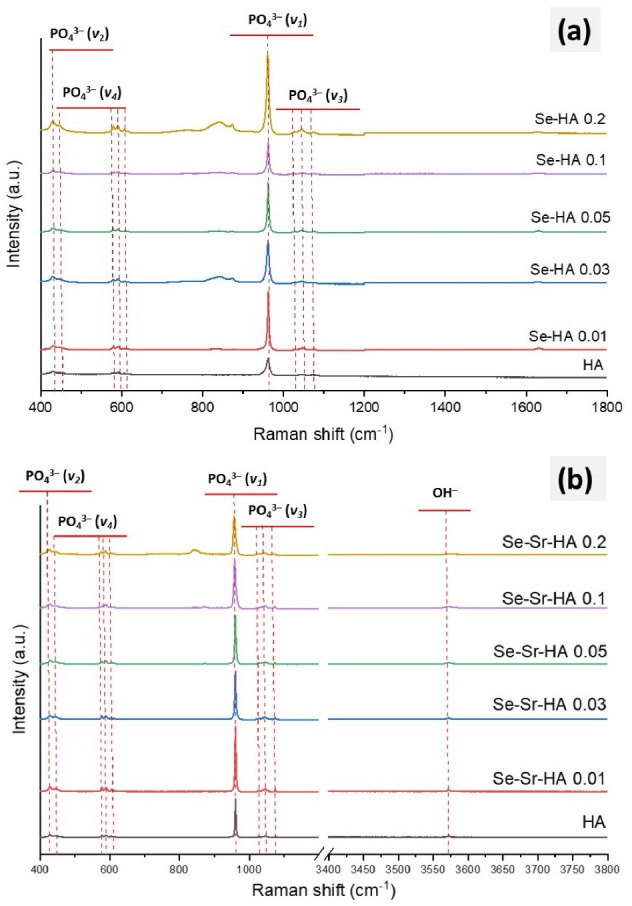
Raman spectra of sintered Se-HA (**a**) and Se-Sr-HA (**b**) at different molar ratios of Sr/(Sr + Ca) and Se/(Se + P).

In order to confirm phosphate substitution with selenite ions, Raman studies were carried out (Figure 3a). Pure HA showed the characteristic bands of hydroxyapatite attributed to different functional groups. Two weak peaks at 1046 cm^−1^ and 1054 cm^−1^ and one medium peak at 1076 cm^−1^ were characteristic of the triply degenerated stretching mode (ν_3 asym._) of the O-P-O bond in a PO_4_^3−^ group. A very sharp peak observed at 960 cm^−1^ was attributed to the stretching mode (ν_1 sym._) of a tetrahedral PO_4_^3−^ group [34]. The peaks at 580 cm^−1^ and 594 cm^−1^ were attributed to triply degenerated bending modes (ν_4_), while 447 cm^−1^ and 428 cm^−1^ were ascribed to doubly degenerated (ν_2_) bending modes of phosphate [29]. All of these bands were present in the Se-HA sample; however, a new peak observed at 830 cm^−1^ was attributed to the symmetrical stretching mode of selenite group (SeO_3_^2−^) in Se-Sr-HA 0.2 [27,34].

All characteristic peaks of hydroxyapatite were also observed in the Raman spectrum of Se and Sr co-substituted HA (Figure 3b). However, Se-Sr co-substituted HA with the highest Se/(Se + P) ratio (Se-Sr-HA 0.2) showed the characteristic peak of the symmetrical stretching mode of the selenite group (SeO_3_^2−^). This might be an indication of some extra-framework selenite phase. This peak was absent at lower Se/(Se + P) and Sr/(Sr + Ca) loadings. Furthermore, with increasing Sr/(Sr + Ca) and Se/(Se + P) ratios, a general shift toward a higher wavenumber in the Raman spectrum was observed. For example, there was a shift in phosphate peak position (ν_1_) from 956 cm^−1^ (in HA) to 960 cm^−1^ (in Sr-Se-HA 0.2), while the phosphate peak ν_3_ shifted from 1074 to 1068 cm^−1^. Similarly, ν_2_PO_4_ shifted from 428 to 425 cm^−1^ with an increasing concentration of selenium and strontium. Thus, FTIR in combination with Raman spectroscopy confirmed the formation of co-substituted HA. However, at higher Se/(Se + P) ratios, in Se-Sr-HA 0.2, extra-framework selenite phases became evident.

#### 2.1.4. XRF Spectrscopy

Table 2 shows the molar concentrations of different elements and the ratio of calcium, phosphorus, strontium, and selenium ions measured through semi-quantitative analysis of both Se-HA and Se-Sr-HA samples. All Se-HA samples showed a Ca/(Se + P) ratio between 1.67 and 1.76, which was slightly higher than the stoichiometric ratio in pure hydroxyapatite, whilst the measured (Ca + Sr)/(P + Se) ratio in Se-Sr-HA was between 1.53 and 1.64, which was slightly lower than that of pure HA. The slight difference might be due to the semi-quantitative mode of analysis.

#### 2.1.5. SEM

**Figure 4:**
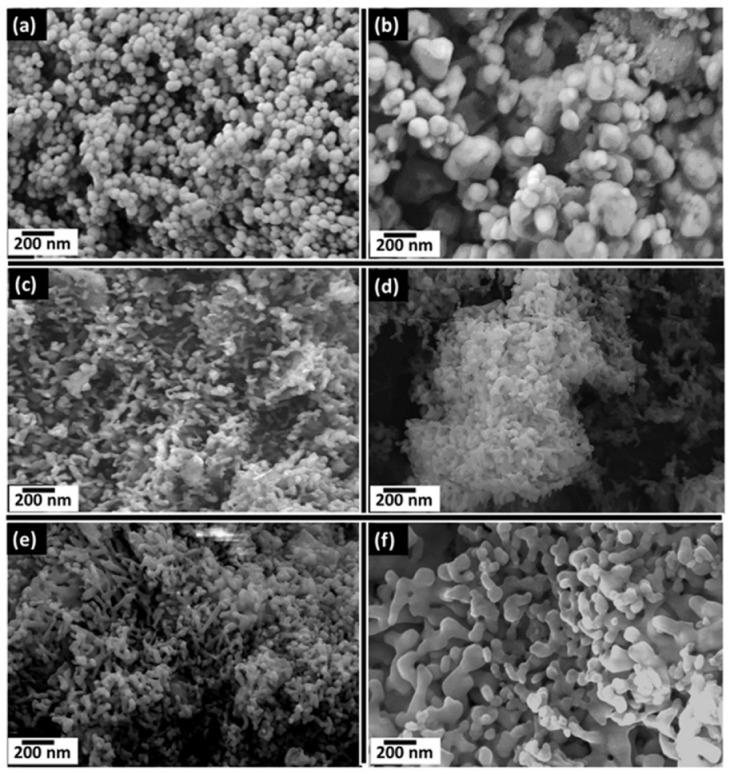
SEM images of HA (**a**), Se-Sr HA 0.01 (**b**), Se-Sr-HA 0.03 (**c**), Se-Sr HA 0.05 (**d**), Se-Sr HA 0.1 (**e**), and Se-Sr HA 0.2 (**f**) powders sintered at 900 °C.

Scanning electron microscopy (SEM) micrographs of pure HA, Se-Sr HA 0.01, Se-Sr HA 0.03, Se-Sr HA 0.05, Se-Sr HA 0.1, and Se-Sr HA 0.2 samples sintered at 900 °C are shown in Figure 4. It is obvious from the micrographs that ion substitution affected the morphology of hydroxyapatite. Pure HA had uniform spherical morphology with particle size in the range of 50–200 nm. The particle size was increased in Se-Sr-HA to 0.01, and the samples were no longer homogeneous. At higher Sr/(Sr + Ca) and Se/(Se + P) ratios, it was observed that the sintering process resulted in the diffusion of particles to form a rod-like irregular structure. In Se-Sr-HA 0.05, more aggregation was observed compared to other samples.

#### 2.1.6. Ion Release Study

**Figure 5:**
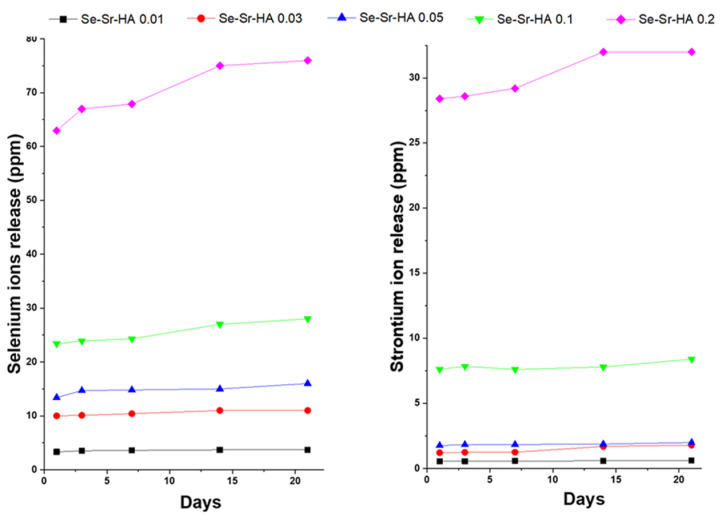
Release profile of strontium and selenium ions from Se-Sr-HA samples immersed in simulated body fluid (SBF) measured by using inductively coupled plasma (ICP-OES).

Figure 5 shows the release profile of SeO_3_^2−^ and Sr^2+^ ions from Se-Sr co-substituted HA under dynamic conditions in simulated body fluid (SBF) solution at 37 °C over a period of 21 days. A burst release of selenium ions was observed in the first 24 h in all Se-Sr-HA samples followed by a steady-state release, indicating long-term sustained release.

#### 2.1.7. Zeta Potential

The zeta potential measurements of HA, Se-HA, Sr-HA, and Se-Sr-HA were performed in ethanol, and the results are given in Table 3. As shown, both selenium and strontium ions changed the surface charge of hydroxyapatite. Strontium substitution resulted in an increase in positive surface charge, while selenium substitution led to a decrease in surface charge. The reason is that strontium (electronegativity 0.95) is more electropositive compared to calcium (electronegativity 1.0), and therefore, its substitution leads to an increase in surface charge [35]. Positive zeta potential increases the solubility of the nanoparticles and may lead to aggregation. However, it also promotes the adsorption of negatively charged proteins on the surface and improves the efficacy of imaging, gene transfer, and drug delivery [21]. Selenium ion reduces the surface charge of HA due to its high electronegativity (2.55) compared to phosphorus (2.19), which facilitates the deposition of Ca^2+^ ions on the surface and enhances the bioactivity [36].

### 2.2. Biological Testing

#### 2.2.1. In Vitro Bioactivity

**Figure 6:**
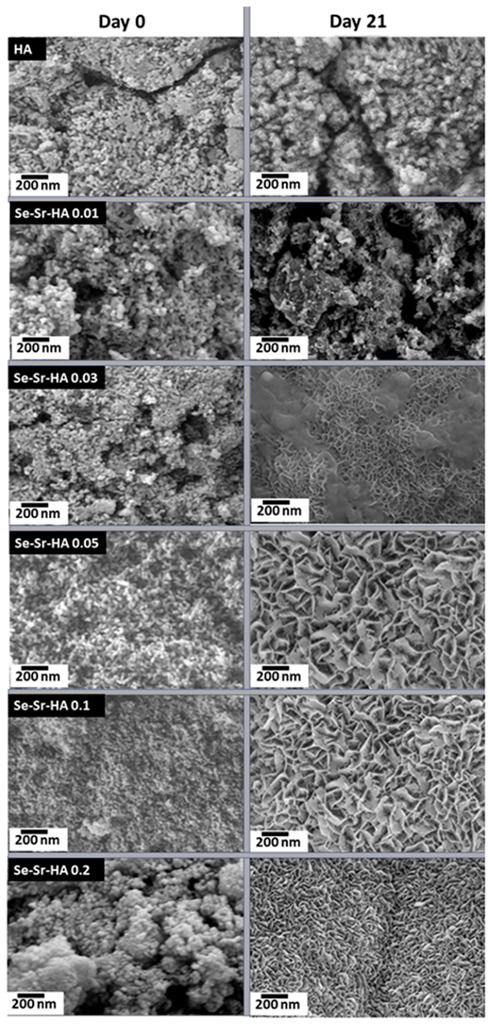
Selenium and strontium co-substituted HA samples before and after soaking in physiological medium (SBF) for 21 days under dynamic conditions at 37 °C.

The effect of metal substitution on bioactivity (defined here as the ability of the material to form CaP on their surface) was studied by soaking the discs in SBF, which is an acellular solution with the inorganic component of blood plasma for a period of 21 days. Figure 6 shows that an apatite layer was formed on all powdered samples sintered at 900 °C. Small apatite crystals were observed on the surface of discs comprising pure HA and Se-Sr-HA 0.01. However, in Sr-Se co-substituted HA with higher Se and Sr ratios, a discernible three-dimensional layer completely covered the surface [37]. The apatite layer was highly dense and thick on the surface of the discs with some precipitation inside the pores. The micrographs show that co-substituted HA was highly bioactive compared to pure HA. Thus, the addition of strontium increased the bioactivity significantly, as also reported in the literature [38].

#### 2.2.2. Antibacterial Activity

**Figure 7:**
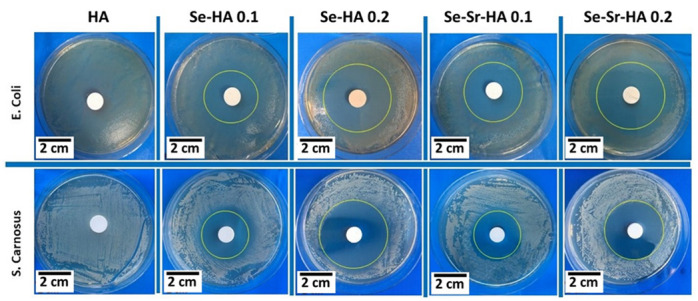
Zone of inhibition test, using disc diffusion assay against Gram-negative (*E. coli*) and Gram-positive (*S. carnosus*) bacteria. The inhibition zones around the Se-Sr-HA discs are clearly shown compared to those of positive control (PC).

The antibacterial effect of pure HA, Se-HA 0.1, Se-HA 0.2, Se-Sr-HA 0.1, and Se-Sr-HA 0.2 was investigated by the antibacterial disc diffusion test (Figure 7). The antibacterial effect on agar was due to direct surface contact between selenium containing HA surface and the bacterium. The antibacterial effect was studied against two strains of bacteria, Gram-negative bacteria (*Escherichia coli*) and Gram-positive bacteria (*Staphylococcus carnosus*). Pure HA did not show any inhibition against both bacteria types. However, the zone of inhibition was observed in all Se-substituted and Se-Sr co-substituted hydroxyapatite samples. Se-Sr-HA 0.1 showed better inhibition against *E. coli* compared to *S. carnosus*, while Se-Sr-HA 0.2 showed almost similar inhibition against both bacteria species [22].

**Figure 8:**
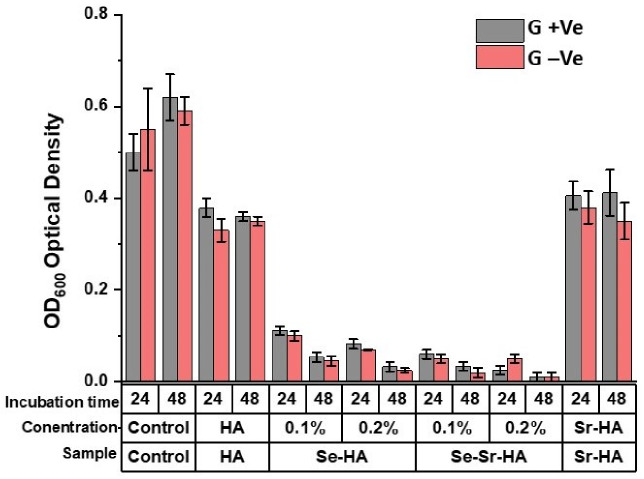
Bacterial turbidity (OD600) study with HA, Se-HA, and Se-Sr-HA samples by the indirect method (0.1 mg in 10 mL PBS) after 24 and 48 h of incubation against Gram+ (G+) and Gram−(G−) bacterium (Cell suspension without HA was the negative control). Data represent mean values ±SDT of *n* = 5 experiments.

The data obtained from the turbidity assay is presented in Figure 8. The optical density (OD) values of Se-HA and Se-Sr-HA samples were reduced in both types of bacteria (*S. carnosus* and *E. coli*) compared to control values (pure HA and Sr-HA). The results showed that the bacterial activity was inhibited, when the Se-HA and Se-Sr-HA supernatants were added to the bacterial suspension. Moreover, the selenium ion release increased with enhanced molar concentration of Se in Se-HA and Se-Sr-HA, Se/(Se + P) from 0.1 to 0.2. The results also revealed that the antibacterial activity of the samples was comparatively more increased, when the interaction time of extracts with bacterial suspension increased from 24 to 48 h. The decreased OD values of Se-HA and Se-Sr-HA samples compared to the control confirmed the growth restriction of both bacterial species (*S. carnosus* and *E. coli*) by the release of selenium ions.

#### 2.2.3. Cytotoxicity Assay (Indirect Method)

**Figure 9:**
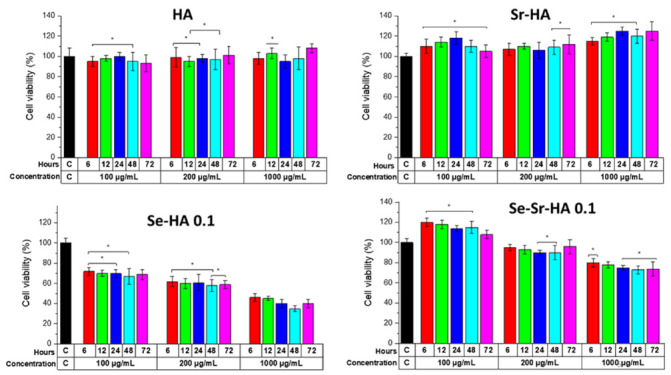
Osteoblast-like human cell (MG-63) response to three different concentrations of supernatants (100 μg·mL^−1^, 200 μg·mL^−1^, and 1000 μg·mL^−1^) of HA, Se-HA, Se-Sr-HA, and Sr-HA taken at different time points (6, 12, 24, 48, and 72 h) measured by WST-8 up to 2 days of culture. Tissue culture plates made up of polystyrene (PS) were used as control (C). The resultant cell viability for each material was normalized against the viability of cells on PS at a particular day (data represent the mean ± standard deviation of two individual experiments each performed in pentaplicate) (* *p* < 0.05).

The cell viability test was performed on the human osteosarcoma cell line (MG63) by the indirect method [39] to evaluate the biological effects of Se and Sr substitution on hydroxyapatite (Figure 9). Three different molar concentrations were investigated for 72 h: 100 µg·mL^−1^, 200 µg·mL^−1^, and 1000 µg·mL^−1^.

## 3. Discussion

Hydroxyapatite is used widely in numerous biomedical and dental applications in the form of granules, fillers, composites, drug carriers, and coatings. The introduction of a biomaterial or an implant in the body, especially in open-fractured bones and joint revision surgeries, is always associated with a risk of bacterial infection [40]. Hence, the main objective of this study was to synthesize a new family of hydroxyapatite bioceramics with inherent antibacterial properties without sacrificing bioactivity and biocompatibility. In order to achieve this, different percentages of selenium and strontium ions were co-substituted in HA by a wet precipitation method. Physicochemical characterization confirmed the substitution of both ions in the hydroxyapatite framework. The crystalline structure of HA was maintained during the substitution reactions. The XRD results further showed that selenium ions were substituted into the HA crystal lattice in the absence of a secondary phase for samples with Se/(Se + P) up to 0.1. In Se-Sr-HA 0.2, some new peaks were observed due to high selenium loading, which were assigned to calcium selenite hydrate (CaSeO_3_·H_2_O, PDF 35-0883). It was obvious that the crystalline phase may change from HA to calcium selenite hydrate with the enhancement of selenite ion concentration. The HAp remained highly crystalline also with combined Sr^2+^ and selenite substitution. The lattice parameter data showed that the substitution of selenium and strontium ions into the HA structure caused an increase in the value of both ‘***a***’ and ‘***c***’ constants. Moreover, no secondary phase of strontium was observed during substitution reaction, which indicated the successful incorporation of strontium in the HA framework. Thus, the XRF data in combination with XRD results prove the SeO_3_^2−^ and Sr^2+^ substitution in the HA crystal structure. If the selenium ions were adsorbed on the crystal surface, the content of selenium would be considerably decreased (soluble selenium salt would be removed during the washing step). The selenium and strontium contents obtained in Se-HA and Se-Sr-HA samples were significantly lower than the nominal values. It appears that the attempted replacement of PO_4_^3−^ ions with such large loading of SeO_3_^2−^ ions was not viable; excess selenium ions remained in the solution and were removed during the washing process. The compositional analysis of Se-Sr-HA by EDS also confirmed the presence of selenium and strontium in the samples (see Supplementary Data, Figure S1). On the basis of XRF, EDS, and XRD results, it could be confirmed that SeO_3_^2−^ and Sr^2+^ ions were successfully substituted in the HA lattice.

Morphological studies showed that substitution also affected the shape of the HA nanoparticles. A change in morphology from spherical to plate-like structure with increasing agglomeration was observed with higher Se and Sr ion concentration. This increase in agglomeration was due to increasing surface charge on substituted hydroxyapatite, which may lead to the diffusion of nanoparticles at high temperature during the sintering process. Similar results have been reported by Zhang et al. [30], who observed the aggregation and formation of needle-like crystals in selenium doped hydroxyapatite. Inductively coupled plasma (ICP-OES) results showed that there was a steady release of both ions from HA in Se-Sr-HA with molar ratios 0.01, 0.03, 0.05, 0.1, and 0.2 in the physiological medium during the 21 days of immersion. However, a burst release of both ions was observed in Se-Sr-HA 0.2, which might be due to the presence of selenium as an additional phase (calcium selenite hydrate, CaSeO_3_·H_2_O). Selenium ions released in the physiological medium play an important role in the antibacterial activity. The results revealing antibacterial properties were in good agreement with ion release data. SeO_3_^2−^ ions released from Se-Sr-HA samples were within the concentration range of 4–76 ppm, which has been proven to induce significant antibacterial properties against Gram-positive and Gram-negative bacterial strains [22]. As evident from Figure 5, the amount of selenium released from Se-Sr-HA 0.2 was higher than that from other samples at all time points, and therefore, Se-Sr-HA 0.2 showed higher antibacterial activity.

Raman spectroscopy also confirmed the substitution of selenium tetrahedron in place of tetrahedral phosphate and incorporation of strontium ions. Peak shifts in the Raman spectrum were due to the change in molecular mass and bonding forces during substitution reactions. As reported by O′Donnell et al. [41], the frequency of Raman bands depends on lattice vibrations, which in turn depend on the mass of atoms/ions and forces between atoms according to Szigeti equation:

$$f=\frac{1}{c}\sqrt{\frac{k}{µ}}$$

where *c* is the velocity of light, *k* is the bond force constant (N·m^−1^), and *μ* is the reduced mass of the two bonding atoms (*m*_1_*m*_2_/(*m*_1_ + *m*_2_)) [41].

Although complexes encompassing selenium have been extensively used in the drug and food industry, some findings gave attention to the biocompatibility and antibacterial properties of selenium-substituted HA particles [42]. Our aim was to reduce the toxicity of Se-HA by co-substituting strontium ions in HA. It was apparent that compared with pure HA and Se-HA, Se-Sr-HA resulted in lower cytotoxicity with cell viability reaching 105% and 120% at the concentrations of 200 and 100 μg/mL, respectively, when cells were incubated with the supernatant of Se-HA-0.1 nanoparticles. It was considered that Sr^2+^ might have direct interaction with the calcium-sensing receptor in MG63 cells to activate mitogenic signals in the protein kinase c/d signaling pathways causing enhanced cell division [43].

## 4. Materials and Methods

### 4.1. Materials

Calcium nitrate tetrahydrate (Ca(NO_3_)_2_.4H_2_O, 98.5%), diammonium hydrogen phosphate ((NH_4_)_2_HPO_4_, 99.5%, sodium selenite ((Na_2_SeO_3_), 99%), strontium nitrate (Sr(NO_3_)_2_, 99.99%), and ammonium hydroxide ((NH_4_OH), 28–30%) were analytical grade chemicals purchased from Sigma Aldrich (Dorset, UK).

#### Synthesis of Se-HA and Se-Sr-HA

The Se-HA with Se/(Se + P) at molar ratios of 0, 0.01, 0.03, 0.05, 0.1, and 0.2 were synthesized by a wet-precipitation method. First, 500 mL of 0.5 M (NH_4_)_2_HPO_4_ and 0.5 M Na_2_SeO_3_ solution were mixed with 500 mL of 0.835 M Ca(NO_3_)_2_.4H_2_O solution. The pH value of Ca(NO_3_)_2_.4H_2_O and (NH_4_)_2_HPO_4_ precursor solutions were adjusted to 11 and 9.5, respectively, by adding 1 M NH_4_OH solution. The Ca/(Se + P) ratio was maintained at 1.67. After mixing of the solutions, the pH value was maintained at 10.75 by adding NH_4_OH, and the final solution was stirred for 16 h at 400 RPM followed by the aging of precipitates for 24 h. The obtained precipitates were washed with de-ionized water (three times) and then centrifuged. The samples were dried at 80 °C in an oven and sintered at 900 °C for 3 h (at the heating rate of 5 °C min^−1^).

Se-Sr-HA at molar ratios of Sr/(Sr + Ca) and Se/(Se + P) at 0, 0.01, 0.03, 0.05, 0.1 and 0.2 were also prepared following the same procedure, using the additional precursor Sr(NO_3_)_2_. Samples were labeled as Se-Sr-HA *x* (where *x* = 0, 0.01, 0.03, 0.05, 0.1, and 0.2).

### 4.2. Physiochemical Characterization

#### 4.2.1. XRD Analysis

XRD was conducted on a Bruker D8 Advance, using Bragg–Brentano parafocusing geometry under a load of 1.6 kW (40 mA and 40 kV). Data were collected for the 2θ range from 20° to 70° (step size of 0.01° and count time 1 s per step) using Cu-Kα radiation monochromated by 20 micron nickel filter, a 0.3 degree divergence slit, and 3 degree receiving slit. The diffraction patterns were compared with the standard diffraction pattern of HA (PDF 09-0432). Collected data were qualitatively examined, using the Bruker diffract+ and EVA search-match software (search matched against the ICDD database). The lattice parameters of HA were obtained by Rietveld refinement using software TOPAS V5 Bruker AXS (Karlsruhe, Germany). For the refinement, the HA structure ICSD #26204 was applied [44].

#### 4.2.2. Fourier Transform Infrared Spectroscopy (FTIR)

Substituted HA samples were analyzed by the FTIR, using a Shimadzu IRAffinity-1S Shimadzu Corp (Tokyo, Japan) in ATR mode. The samples were scanned in the transmittance mode at wavenumbers ranging from 4000 to 400 cm^−1^ and the resolution was 4 cm^−1^.

#### 4.2.3. Raman Spectroscopy

Raman spectroscopy was used to investigate the functional groups present in substituted hydroxyapatites. The spectrum was taken using a LabRAM HR800, Horiba Jobin Yvon (Kyoto, Japan) instrument, using a 532 nm laser line. The samples were scanned in the range of 300–1200 cm^−1^ at 30 s for each scan, and the final spectrum was produced by collecting and summing up 3 scans.

#### 4.2.4. Scanning Electron Microscopy-Energy-Dispersive X-ray Spectroscopy (SEM-EDS)

The structural morphology of the synthesized samples was studied, using Field Emission Scanning Electron Microscope (FESEM), model LEO 435VP from Carl Zeiss™ AG (Jena, Germany). The images were taken at an energy of 10–15 kV. Prior to imaging, the samples were gold-coated, using sputter Q150/ S, Quorum Technologies™ (Darmstadt, Germany). The elemental compositional analysis was performed by EDS (LEO 435VP, Carl Zeiss™ AG (Jena, Germany) at 15 kV.

#### 4.2.5. X-ray Fluorescence Spectroscopy (XRF)

For compositional analysis by X-ray fluorescence (XRF), the samples were prepared and reported in accordance with ISO standard 12677. The substituted HA samples were ignited at 1200 °C for 1 h to examine the loss on ignition. Then, approximately 1.5 g of powdered sample was mixed with 7.5 g of Li_2_B_4_O_7_ (lithium tetraborate) and heated in a platinum crucible at 1025 °C for 20 min followed by heating at 1200 °C for 5 min. Then the glass melt was cast into a glass disc to obtain a glassy bead, which was analyzed by utilizing a Panalytical AXIOS,WD-XF Sequential Spectrometer on its IQ+ standardless semiquantitative package.

#### 4.2.6. Ion-Release Profile

To investigate the selenium and strontium ion release from prepared Se-HA and Se-Sr-HA samples, 75 mg of powder sample was dispersed in SBF solution (50 mL) for different time intervals (day 1, day 3, day 7, day 14, and day 21) in a shaker incubator at 37 °C at 200 RPM. Selenium and strontium ions released in the medium under dynamic conditions were measured, using ICP-OES (inductively coupled plasma/optical emission spectrometry) from IRIS Advantage, Thermo Jarrell Ash. Approximately 1 g of the sample was dissolved in a 5% HNO_3_ solution and heated gently to ensure complete dissolution. The solution was made up to 50 mL volumetrically and analyzed by ICP-OES IRIS Advantage, Thermo Jarrell Ash (Waltham, MA, USA) against a calibration traceable under ISO: 17025 guidance.

#### 4.2.7. Zeta Potential

To measure the zeta potential, the Zetasizer Nano zsp Malvern Panalytical GmbH (Worcestershire, UK) was used. The analyzed suspensions (powder samples in absolute ethanol) were diluted to the ratio of 0.1 g·L^−1^ in particles. Three measurements per suspension at standard pH were taken at a maximum of 100 runs each and averaged. After each measurement, the cell was flushed out with ethanol again.

### 4.3. Biological Studies

#### 4.3.1. In Vitro Bioactivity in Simulated Body Fluid (SBF)

The acellular in vitro bioactivity of HA, Se-HA, Se-Sr-HA, and Sr-HA samples was investigated in SBF following the protocol reported by Kokubo et al. [45]. The SBF preparation method is described in the Supplementary Information section (S2) and the chemicals used to prepare SBF are listed in Table S1. The samples (pellets, 13 mm diameter) were immersed in SBF medium (50 mL) and placed at 37 °C in an oven for 21 days. SBF was changed after every 48 h. After 21 days, the samples were taken out, rinsed with de-ionized water, and air-dried. The formation of an apatite layer on the sample surface was investigated by scanning electron microscopy (SEM).

#### 4.3.2. Antibacterial Studies

##### Disc Diffusion Method

The antibacterial effect of samples was investigated by the antibacterial disc diffusion test [46,47], using unsubstituted HA as a control. The samples were sterilized in a heating oven for 2 h at 160 °C. Then, 20 µL of LB-media containing *Escherichia coli* or *Staphylococcus carnosus* at an optical density of 0.015 (OD_600_) were spread homogeneously on agar plates. Then, HA disc samples were placed on these agar plates and incubated at 37 °C for 24 h. The zones of inhibition for each plate were measured after 24 h of incubation (each test was performed in triplicate).

##### Turbidity Test Using Optical Density Measurements

The effect of ion release from substituted HA powdered samples was examined via an indirect method [48]. The powdered samples were immersed in phosphate-buffered saline (PBS) for 24 h, and the supernatant extract was co-cultured with bacteria. The optical density (OD) of this medium was measured and reported in terms of turbidity. Higher optical density values were associated with greater turbidity of the bacterial suspension developed from bacterial growth during the incubation course.

#### 4.3.3. In Vitro Cytocompatibility (Indirect Method)

##### Extract Preparation

The in vitro cell biocompatibility of samples was investigated, using human osteosarcoma cell line MG-63 (Sigma-Aldrich). The cytotoxicity tests were performed via the indirect method [49] on powdered samples (0.99 g) sintered at 900 °C. Samples were soaked in Dulbecco′s Modified Eagle′s Medium (DMEM) (9.9 mL), shaken well, and incubated at 37 °C. Then, supernatants of 3 different concentrations (100, 200, and 1000 μg·mL^−1^) were taken at different time points (6, 12, 24, 48, and 72 h). The cells were cultured in T75 to 90% confluency, using 1 mL (300,000 cells in each well) of (DMEM) supplemented with 10% fetal bovine serum (Sigma-Aldrich) and 1% penicillin–streptomycin (Sigma-Aldrich) and incubated for 48 h at 37 °C and 5% CO_2_.

##### Cell Viability (WST-8) Assay

The cell viability was measured by evaluating the mitochondrial activity in cells by the WST-8 assay. The medium was removed from wells and completely rinsed two times with PBS. Then, 100 μL of new DMEM medium containing WST-8 (1 vol%) was added to each well. After 2 h incubation at 37 °C, the absorbance was logged at 450 nm on a microplate reader (Anthos-Phomo, Germany). The positive control absorbance was taken as 100% viability value, while DMEM + 1% WST-8 was taken as blank. The cell viability (%) was computed with following equation:

$$Cell\;viability=\frac{OD\;\left(test\right)-OD\left(blank\right)}{OD\left(positive\;control\right)-OD\left(blank\right)}\times 100\%.$$

## 5. Conclusions

In this manuscript, a novel homogeneous selenium and strontium co-substituted hydroxyapatite powder with antibacterial properties and excellent biological activity was prepared by a simple wet precipitation method. The characterization results revealed that selenium and strontium ions were successfully substituted for tetrahedral phosphate and calcium ions, respectively, in hydroxyapatite during the synthesis. However, the level of ion substitution was lower than theoretical predictions. The stoichiometric Ca/P ratio of 1.67 was largely maintained during synthesis, and no secondary calcium phosphate phases were detected by XRD. Zeta potential results showed that the HA surface became more positive after Sr^2+^ substitution, while SEM showed that the HA particle morphology was changed from uniform spheres to a plate-like aggregated structure. The ion release study in SBF solution showed that there was a steady ion release of both strontium and selenium under dynamic conditions, which is very important for surgical applications, where a slow and continuous release of the antimicrobial agent is a challenge. The co-substituted HA was effective against both Gram-positive and Gram-negative bacteria with almost zero survival rate. Cell viability assay (WST-8) showed that cytotoxicity of selenium was offset by strontium, which possesses excellent biocompatibility, and therefore, Se-Sr-HA was cytocompatible in contact with MG-63 osteosarcoma cell line. Thus, the selenium and strontium co-substituted hydroxyapatite reported here offers an excellent novel bioceramic for implant coatings, dental filler, and gene carrier applications.

## Figures and Tables

**Table 1:** Lattice parameters of HA and Se-Sr-HA with different Se/(Se + P) and Sr/(Ca + Sr).

Sample	*a*-Axis (Å)	*c*-Axis (Å)	Unit Cell Volume (Å)^3^
HA	9.4420	6.8612	528.0
Se-Sr-HA 0.01	9.4347	6.8893	530.4
Se-Sr-HA 0.03	9.452	6.9041	532.2
Se-Sr-HA 0.05	9.4706	6.9232	533.8
Se-Sr-HA 0.1	9.4869	6.942	535.1
Se-Sr-HA 0.2	9.5062	6.9609	536.9

**Table 2:** Expected and measured elemental composition of HA, Se-HA, and Se-Sr-HA samples at different Se and Se/Sr molar ratios after sintering at 900 °C.

Samples	Calcium	Phosphorus	Selenium	Strontium	(Ca + Sr)/(P + Se) Ratio	
	(moles)	(moles)	(moles)	(moles)	Calculated	XRF
HA	1.09	0.65	0	0	1.67	1.67
Se-Sr-HA 0.01	1.03	0.63	0.0015	0.0067	1.70	1.64
Se-Sr-HA 0.03	0.99	0.62	0.0036	0.0261	1.67	1.64
Se-Sr-HA 0.05	0.96	0.60	0.0045	0.0338	1.67	1.63
Se-Sr-HA 0.1	0.94	0.59	0.0117	0.0386	1.67	1.63
Se-Sr-HA 0.2	0.82	0.55	0.0441	0.0897	1.67	1.53
**Se-HA Samples**					**Ca/(P + Se)**	
Se-HA 0.01	1.09	0.65	0.003	—	1.67	1.67
Se-HA 0.03	1.07	0.63	0.004	—	1.67	1.67
Se-HA 0.05	1.03	0.60	0.004	—	1.67	1.69
Se-HA 0.1	1.02	0.56	0.018	—	1.67	1.75
Se-HA 0.2	1.03	0.53	0.051	—	1.67	1.76

**Table 3:** Zeta potential measurement for HA, Sr-HA, Se-HA, and Se-Sr-HA nanoparticles in pure ethanol.

Particles	Zeta Potential ± SD [mV]
HA	24 ± 3
Se-HA 0.1	12 ± 2
Sr-HA 0.1	31 ± 3
Se-Sr-HA 0.1	18 ± 2

## Data Availability

Not applicable.

## Supplementary Materials

The following are available online at https://www.mdpi.com/article/10.3390/ijms22084246/s1. Figure S1: EDS spectra of Se-Sr-HA 0.01, Se-Sr-HA 0.03, Se-Sr-HA 0.05, Se-Sr-HA 0.1, & Se-Sr-HA 0.2, Table S1: Recipe for preparation of SBF according to Kokubo 1.
