# Supplementary material for: Synthesis, Characterization, Antibacterial Properties, and In Vitro Studies of Selenium and Strontium Co-Substituted Hydroxyapatite
Source: Int J Mol Sci. 2021 Apr 19;22(8):4246. doi: 10.3390/ijms22084246 (PMC8072711; doi:10.3390/ijms22084246)
Supplement: Supplementary file 1 [file ijms-22-04246-s001.zip › ijms-1159369-supplementary.pdf]

Supplementary Information

S 1: EDS data

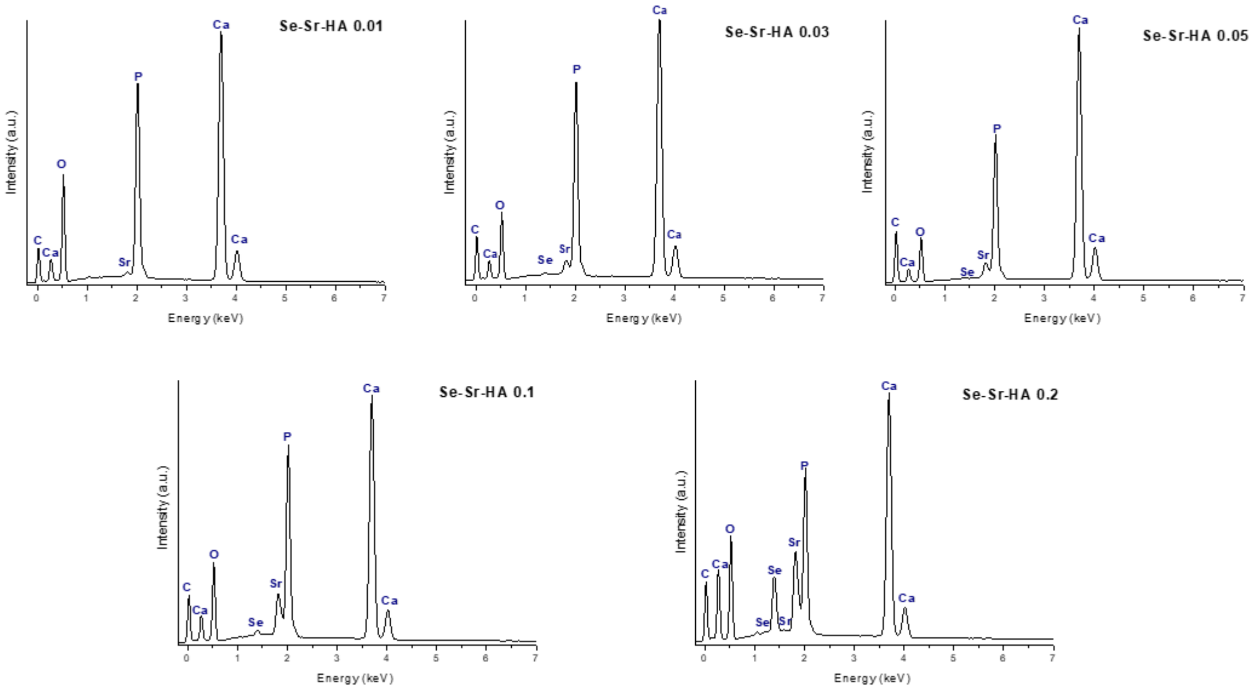

Figure S1: EDS spectra of Se-Sr-HA 0.01, Se-Sr-HA 0.03, Se-Sr-HA 0.05, Se-Sr-HA 0.1, & Se-Sr-HA 0.2

The compositional analysis of selenium strontium co-substituted HA by EDS also confirmed the presence of selenium and strontium in the samples. The EDS results are in good agreement with the compositional data by XRF. By XRF, EDS and XRD results, we believe that SeO_3^{2-} and Sr^{2+} ions were successfully substituted in the HA lattice.

S 2: SBF Preparation

The SBF was prepared using the components in Table S1 according to the Kokubo and Takadama method [45]. At the start of SBF preparation, reagents in the order 1 – 8 (Table S1) were sequentially added and dissolved in 750 mL of distilled water taken from the ELGA DV 25 (PURELAB Option R7BP, Wycombe, United Kingdom) in a container, which was held at a temperature range of 36 °C ± 1 °C whilst being magnetically stirred. Following complete reagents dissolution, distilled water was poured to rise the overall volume to 900 mL. In the following step TRIS (order 9) was added at a temperature range of 36 °C ± 1 °C (1). pH monitoring was performed using a 3510 pH meter (Bibby Scientific Limited, Staffordshire, UK). TRIS was added till the pH was enhanced to 7.30 ± 0.05. Subsequently, TRIS was added in a temperature window of 36 °C ± 0.5 °C until a pH value of 7.45 ± 0.01 was attained. Hydrochloric acid (order 10) was then added to reduce the pH value to 7.42 ± 0.01. In the last phase, distilled water was poured to get a final volume of 1 litre.

Table S1: Recipe for preparation of SBF according to Kokubo [45]

Order	Reagent	Distributor	Purity	Amount
	Distilled water	-	-	≥ 900 mL
1	Sodium chloride (NaCl)	GPR RECAPTURE® VWR International	> 99 %	7.9948 g
2	Sodium bicarbonate ($NaHCO_3$)	ACS reagent Merck KGaA	99.7 %	0.3543 g
3	Potassium chloride (KCl)	EMSURE® Merck KGaA	99.7 %	0.225 g
4	Potassium phosphate dibasic trihydrate ($K_2HPO_4 \cdot 3H_2O$)	ReagentPlus® Merck KGaA	99.7 %	0.231 g
5	Magnesium chloride hexahydrate ($MgCl_2 \cdot 6H_2O$)	ACS reagent Merck KGaA	100 %	0.3303 g
6	Hydrochloric acid (HCl)	AnalaR NORMAPUR® Reag. Ph. Eur. analytical reagent VWR International	1 mole/litre	39 mL
7	Calcium chloride dihydrate ($CaCl_2 \cdot 2H_2O$)		100 %	0.3638 g
8	Sodium sulphate anhydrous (Na_2SO_4)		99.6 %	0.0716 g
9	Tris(hydroxylmethyl)aminomethane	GPR RECTAPUR® VWR International	100 %	6.0568 g
10	Hydrochloric acid (HCl)	Reag. Ph. Eur. analytical reagent VWR International	1 mole/litre	0 – 5 mL
